# Mathematical modeling of multicellular tumor spheroids quantifies inter-patient and intra-tumor heterogeneity

**DOI:** 10.1038/s41540-025-00492-3

**Published:** 2025-02-15

**Authors:** Adam A. Malik, Kyle C. Nguyen, John T. Nardini, Cecilia C. Krona, Kevin B. Flores, Sven Nelander

**Affiliations:** 1 Mathematical Sciences, Chalmers University of Technology, Gothenburg, Sweden; 2 Biomathematics Graduate Program, North Carolina State University, Raleigh, NC USA; 3 Center for Research in Scientific Computation, North Carolina State University, Raleigh, NC USA; 4 Department of Mathematics and Statistics, The College of New Jersey, Ewing, NJ USA; 5 Department of Immunology, Genetics and Pathology, Uppsala University, Uppsala, Sweden; 6 Department of Mathematics, North Carolina State University, Raleigh, NC USA

**Keywords:** Applied mathematics, Computational biology and bioinformatics, Cancer

## Abstract

In the study of brain tumors, patient-derived three-dimensional sphere cultures provide an important tool for studying emerging treatments. The growth of such spheroids depends on the combined effects of proliferation and migration of cells, but it is challenging to make accurate distinctions between increase in cell number versus the radial movement of cells. To address this, we formulate a novel model in the form of a system of two partial differential equations (PDEs) incorporating both migration and growth terms, and show that it more accurately fits our data compared to simpler PDE models. We show that traveling-wave speeds are strongly associated with population heterogeneity. Having fitted the model to our dataset we show that a subset of the cell lines are best described by a “Go-or-Grow”-type model, which constitutes a special case of our model. Finally, we investigate whether our fitted model parameters are correlated with patient age and survival.

## Introduction

Glioma grade IV, or gliblastoma multiforme (GBM) stands as the most prevalent diagnosis among all gliomas. It is notoriously malignant, with the majority of GBM patients succumbing to the disease within a year^1,2^. This outcome can be attributed to pronounced inter- and intra-tumor heterogeneity, coupled with the infiltrative nature of the tumor itself^3^. Glioblastoma, often treated with surgery, radiotherapy, chemotherapy, or a combination, faces high recurrence rates^1^. Surgical challenges arise due to the tumors’ diffuse nature, with cells capable of extensive migration through healthy tissue to infiltrate crucial regions of the brain^2^. Research endeavors span multiple domains, encompassing imaging and surgical approaches^4^, advancements in radiotherapy techniques^5^, exploration of immunotherapeutic avenues^6^, pharmaceutical interventions^7–9^, as well as mathematical and computational modeling^10,11^. These multifaceted efforts underscore the urgent need for innovative approaches to confront the formidable challenges posed by GBM. In this study we adopt a multidisciplinary approach towards GBM by combining large scale multicellular tumor spheroid experiments from patient-derived cells, with novel mathematical modeling and analysis.

A popular experimental model for GBM tumors are 3D cultured multicellular tumor spheroids (MCTSs). They replicate the in vivo environment more faithfully compared to 2D monolayer models^12^. Unlike cells cultured on flat surfaces, those in 3D environments experience crucial cell-cell and cell-extracellular matrix interactions that mimic the natural conditions within or in proximity to a tumor. Consequently, 3D tumor spheroid assays have emerged as invaluable tools in drug screens and therapeutic efficacy evaluation^13,14^.

Many mathematical models have been introduced to describe the properties of GBM^15–24^. For example, the Fisher Kolmogorov-Petrovsky-Piskunov (Fisher-KPP) equation, a type of reaction-diffusion equation,

$$\frac{\partial u}{\partial t}=\nabla \cdot (D\nabla u)+\rho u\left(1-\frac{u}{K}\right),$$

is the simplest partial differential equation (PDE) model that can be used to describe the spatial and temporal in vitro or in vivo spreading of GBM^15–22^. With the aid of the Fisher-KPP equation, Baldock et al.^18^ showed that patients with less invasive GBM tumors would receive more benefit from resection. While previous studies have shown that the Fisher-KPP equation is clinically relevant^16–18,25^, the model assumes an intratumor homogeneous cellular behavior that might not be true in GBM populations^26^. As a result, many PDE models have been introduced to account for the population heterogeneity in GBM populations^24,26–34^.

MCTSs are marked by the presence of heterogeneity. The tumors are often described as having distinct regions, such as a core and an invasive rim^26^. A property that is often seen in MCTS spreading is that the core and the invasive rim expand at different velocities^35^, a phenomenon that cannot be explained by the Fisher-KPP equation. Another property that has been the subject of debate, from both a biological^36–39^ as well as a mathematical^26,33,40,41^ viewpoint, is the “Go-or-Grow” hypothesis. The hypothesis postulates that cells are either migrating or proliferating, and that the two processes are mutually exclusive. This hypothesis requires a more intricate model than the Fisher-KPP equation.

Our novel model involves two distinct populations, whose governing differential equations include reaction, diffusion, and advection terms. This model provides a comprehensive framework to capture the aforementioned complexities of growing tumor spheroids. To validate the efficacy of our model, we compared it to three other PDE models: the Fisher-KPP model with and without advection, as well as a 2-system PDE without advection. Our results demonstrate the superior fit of our proposed model to the data, underscoring its capacity to more accurately represent the observed dynamics. Furthermore, we conducted extensive fitting of our model to a diverse set of patient-derived cell lines, revealing notable heterogeneity between different patients. This exploration not only enhances our understanding of the varying growth patterns but also positions our model as a valuable tool for personalized medicine strategies. Finally, our model’s ability to provide meaningful insights is exemplified by its correlation with patient age and survival. Certain model parameters were found to be indicative of spheroid growth patterns that are associated with patient outcomes, which highlights the potential clinical relevance of our mathematical framework and its capacity to contribute to a more nuanced understanding of growing tumor spheroids. To the best of our knowledge, this is the first time PDE model parameters from MCTS experiments have been connected to patient survival.

**Fig. 1:**
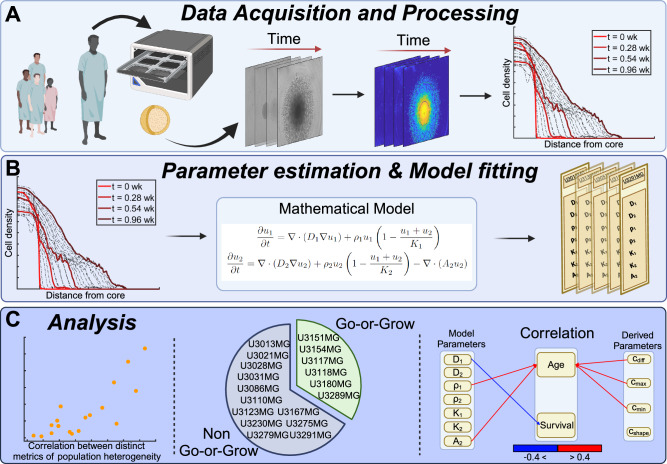
Paper outline and workflow. **A** Image sequences of multicellular tumor spheroids of patient-derived cell cultures are acquired. Images are processed and turned into density profiles describing the cell density as a function of radial distance from the tumor core. **B** Our mathematical model, a system of two PDEs, is fitted to the dataset and model parameters are obtained for each cell line. **C** Our analysis demonstrates a number of insights, including the correlation between wave-parameters and population heterogeneity, identification of cell lines exhibiting Go-or-Grow behavior, as well as the correlation between model parameters and clinical data. This figure was created with BioRender.com.

The organization of this paper is outlined in Fig. 1 and proceeds as follows. The results are presented in the “Results” section (Fig. 1C), where we first present our mathematical model (see the “Mathematical models” section) and compare our model to existing models of the same type (see the “Population heterogeneity and advection improves model fit” section), analyze the fitted models in relationship to cell line heterogeneity (see the “Wave front speeds approximate population heterogeneity” section), determine which cell lines are exhibiting Go-or-Grow behavior (see the “A subset of cell lines exhibit Go-or-Grow behavior” section), as well as investigate the association between model parameters and patient survival (see the “Correlation between patient outcome and mathematical model parameters” section). The “Discussion” section is devoted to a discussion and concluding remarks. Our methodology is presented in the “Methods” section (Fig. 1A), where we describe the data acquisition process (see the “Spheroid invasion assay” section), followed by the data processing steps (see the “Data processing” section). We then describe the parameter estimation procedure (see the “Parameter estimation method” section) (Fig. 1B).

## Results

### Mathematical models

Previous work has shown the clinical relevancy of the Fisher-KPP equation’s parameters^16,18,21,25^. Extending from this equation, we propose a new model while accounting for the “Go-or-Grow” hypothesis to describe populations of phenotypically different subpopulations. Our proposed model is a spatiotemporal system that consists of two PDEs describing cell densities of two populations of cells: reaction-diffusion equation (Eq. (3)) and advection-reaction-diffusion equation (Eq. (4)), we refer to this model as the RD-ARD model. The first population density, ${u}_{1}(\overrightarrow{{\boldsymbol{x}}},t)$, is governed by the reaction-diffusion equation while the second population density, ${u}_{2}(\overrightarrow{{\boldsymbol{x}}},t)$, is a more migrative population and is governed by an advection-reaction-diffusion equation. The model is described as follows:

1 $$\frac{\partial {u}_{1}}{\partial t}=\nabla \cdot ({D}_{1}\nabla {u}_{1})+{\rho }_{1}{u}_{1}\left(1-\frac{{u}_{1}+{u}_{2}}{{K}_{1}}\right),$$

2 $$\frac{\partial {u}_{2}}{\partial t}=\nabla \cdot ({D}_{2}\nabla {u}_{2})+{\rho }_{2}{u}_{2}\left(1-\frac{{u}_{1}+{u}_{2}}{{K}_{2}}\right)-\nabla \cdot ({A}_{2}{u}_{2}),$$

where *D*_1_ and *D*_2_ are the diffusion coefficients, *ρ*_1_ and *ρ*_2_ are the proliferation rates, *K*_1_ and *K*_2_ are the carrying capacities for the first and second subpopulations, respectively, and $\overrightarrow{{\boldsymbol{x}}}={(x,y,z)}^{T}$. In addition, for the second subpopulation, we include an advection term with advection coefficient *A*_2_. We do not consider density-dependent or other non-linear effects in this work, instead we assume that all coefficients are positive real numbers. Cell migration due to diffusion influences both populations, however, only the second population is assumed to be influenced by extrinsic microenvironmental factors that result in the advection term. We want to note that the advection term has been previously used to model the cell migration in GBM^23,26^. Although the sources of these factors are unknown, it has been suggested that it could be due to cells getting attracted by nutrients, oxygen, etc.^26^, or other biases causing directed motion. The growth terms are governed by the logistic growth with the inclusion of competition between cells. The aggregated cell density is the sum of the two subpopulation densities: *u*(*r*, *t*) = *u*_1_(*r*, *t*) + *u*_2_(*r*, *t*). Equations (1–2) describe the time evolution of the two cell subpopulations in three-dimensional space $\overrightarrow{{\boldsymbol{x}}}$ however, our processed data is the one-dimensional averaged cell density as a function of the radial distance from the spheroid center, *r*. To address this discrepancy, we simulate eqs (3–4) using radially-symmetric spherical coordinates

3 $$\frac{\partial {u}_{1}}{\partial t}=\frac{1}{{r}^{2}}\frac{\partial }{\partial r}\left({r}^{2}{D}_{1}\frac{\partial {u}_{1}}{\partial r}\right)+{\rho }_{1}{u}_{1}\left(1-\frac{{u}_{1}+{u}_{2}}{{K}_{1}}\right),$$

4 $$\frac{\partial {u}_{2}}{\partial t}=\frac{1}{{r}^{2}}\frac{\partial }{\partial r}\left({r}^{2}{D}_{2}\frac{\partial {u}_{2}}{\partial r}\right)+{\rho }_{2}{u}_{2}\left(1-\frac{{u}_{1}+{u}_{2}}{{K}_{2}}\right)-\frac{\partial }{\partial r}\left({A}_{2}{u}_{2}\right).$$

The initial conditions, *u*_10_(*r*) and *u*_20_(*r*), for the subpopulations are unknown, however, we observe the total initial cell population *u*_0_(*r*). Therefore, we assume that *u*_10_(*r*) and *u*_20_(*r*) are proportional to the initial total population: *u*_10_(*r*) = *αu*_0_(*r*) and *u*_20_(*r*) = (1 − *α*)*u*_0_(*r*), where *α* is a proportional constant parameter that can be estimated and *u*_0_(*r*) is the cell density data at the initial time point. Similar to previous studies^24,42^, the boundary conditions are taken to be no-flux condition for each subpopulation:

$$\begin{array}{r}\displaystyle\frac{\partial {u}_{1}}{\partial r}(0,t)=\displaystyle\frac{\partial {u}_{1}}{\partial r}(L,t)=0,\\ \displaystyle\frac{\partial {u}_{2}}{\partial r}(0,t)=\displaystyle\frac{\partial {u}_{2}}{\partial r}(L,t)=0.\end{array}$$

Throughout this study, the spatial domain is chosen to be 0 ≤ *r* ≤ 5 mm, and the temporal domain to be 0 ≤ *t* ≤ 1 weeks, as is dictated by the multicellular tumor spheroid experiments conducted.

In addition to the RD-ARD model, Table 1 contains the other three models we test in this study. Note that we compute these three-dimensional models in spherical coordinates to facilitate their comparison to the one-dimensional data. The Reaction-diffusion (RD) model, which has been found to be clinically relevant^16,18,21,25^, assumes that there is a single population of cells which undergoes diffusion and proliferation. The Advection-reaction-diffusion (ARD) model assumes that a single population of cells undergoes advection, diffusion, and proliferation. The reaction-diffusion and reaction-diffusion (RD-RD, or 2-population RD) model describes two populations of cells which both undergo diffusion proliferation; each population has their own parameters for these actions. The initial and boundary conditions for the RD-RD model are equivalent to those presented for the RD-ARD model. For the RD and ARD models, the initial condition is the initial total population density observed in the data, and the boundary condition is similar to that of the RD-ARD model (i.e., a no-flux condition for the homogeneous population).

**Table 1:** Summary of models and sets of parameters to be estimated

Model	Equations	Parameters
RD	$\frac{\partial u}{\partial t}=\nabla \cdot (D\nabla u)+\rho u\left(1-\frac{u}{K}\right)$	*D*, *ρ*, *K*
ARD	$\frac{\partial u}{\partial t}=\nabla \cdot (D\nabla u)+\rho u\left(1-\frac{u}{K}\right)-\nabla \cdot (Au)$	*D*, *ρ*, *K*, *A*
RD-RD	$\frac{\partial {u}_{1}}{\partial t}=\nabla \cdot ({D}_{1}\nabla {u}_{1})+{\rho }_{1}{u}_{1}\left(1-\frac{{u}_{1}+{u}_{2}}{{K}_{1}}\right)$ $\frac{\partial {u}_{2}}{\partial t}=\nabla \cdot ({D}_{2}\nabla {u}_{2})+{\rho }_{2}{u}_{2}\left(1-\frac{{u}_{1}+{u}_{2}}{{K}_{2}}\right)$	*D*_1_, *ρ*_1_, *K*_1_, *α*, *D*_2_, *ρ*_2_, *K*_2_
RD-ARD	$\frac{\partial {u}_{1}}{\partial t}=\nabla \cdot ({D}_{1}\nabla {u}_{1})+{\rho }_{1}{u}_{1}\left(1-\frac{{u}_{1}+{u}_{2}}{{K}_{1}}\right)$ $\frac{\partial {u}_{2}}{\partial t}=\nabla \cdot ({D}_{2}\nabla {u}_{2})+{\rho }_{2}{u}_{2}\left(1-\frac{{u}_{1}+{u}_{2}}{{K}_{2}}\right)-\nabla \cdot ({A}_{2}{u}_{2})$	*D*_1_, *ρ*_1_, *K*_1_, *α*, *D*_2_, *ρ*_2_, *K*_2_, *A*_2_

### Population heterogeneity and advection improves model fit

**Fig. 2:**
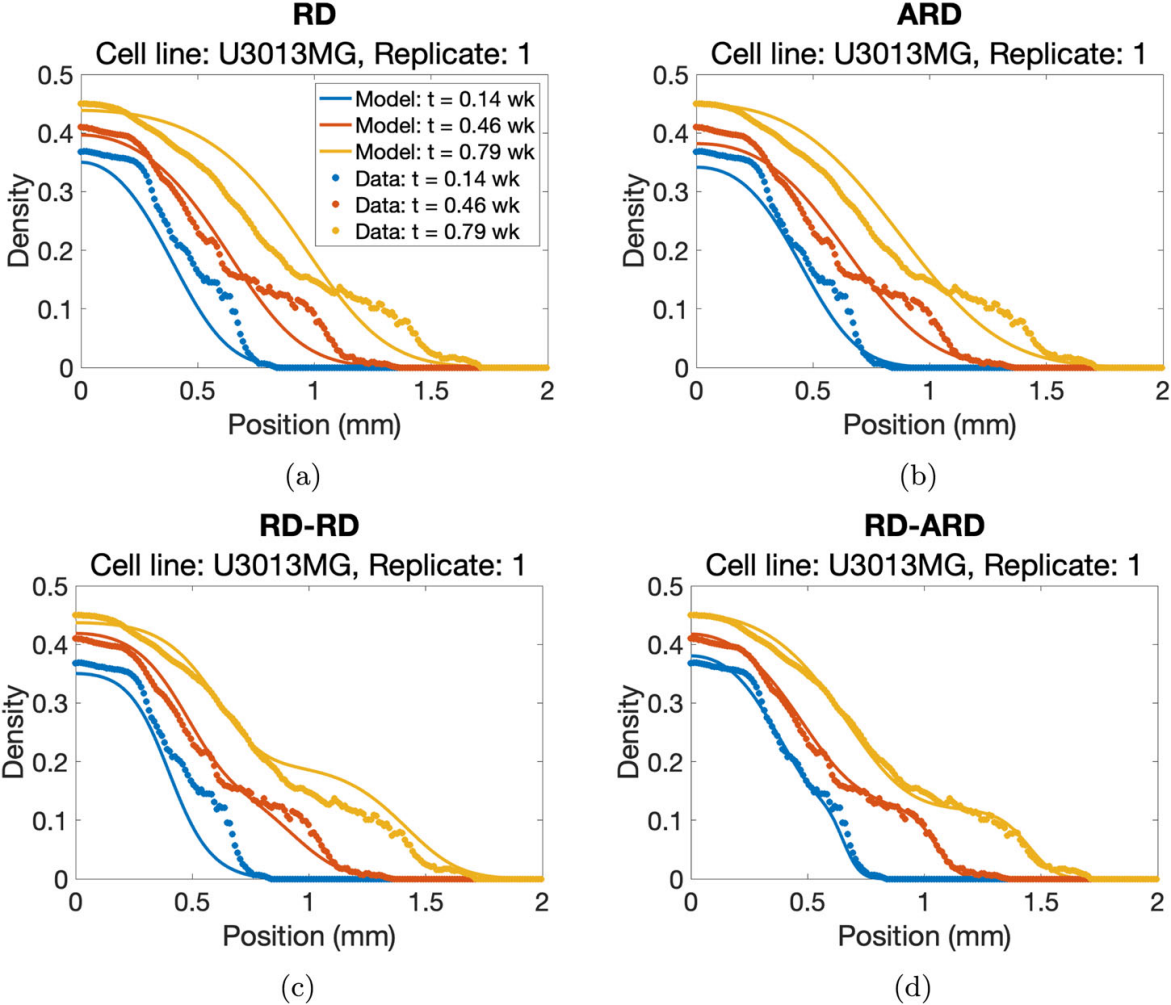
A visualization of fitting results using 4 models. **a** RD, **b** ARD, **c** RD-RD (or 2-population RD), and **d** RD-ARD models for the first replicate of cell line U3013MG. We plot the total cell density data against the location for different time point as dotted color curves. The solid color curves represents the model fittings. Time is measured in weeks.

**Fig. 3:**
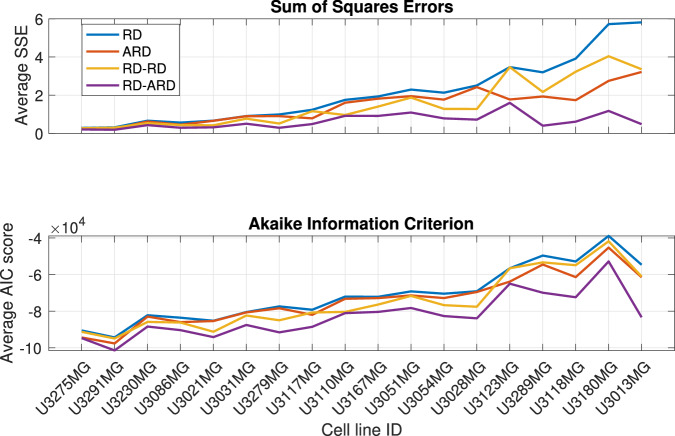
Model performance comparison. Model performance comparison using sum of squares error (top) and Akaike Information Criterion (bottom).

We estimated the parameters for all four models against a total of 136 datasets from 18 different patient-derived cell lines. The full description of the parameter estimation procedure can be found in the “Parameter estimation method” section. The best-fit simulations from all models are plotted against the first replicate of cell line U3013MG (Fig. 2, see Supplementary Figures 1–4 for additional representative results for other cell lines). This figure shows that the RD-ARD model best captures the dynamics in the data, especially the observed “hump”, i.e., the invasive front of cells at cell densities around 0.1. On the other hand, other models, especially the RD and ARD models, fail to capture this. Similar observations can be found for other replicates of cell line U3013MG (see GitHub repository provided in the Code availability statement). We summarized the performance of all four models in describing each dataset by computing their sum of squares error (*SSE*) and Akaike information criterion (*AIC*) scores (Fig. 3). The RD-ARD model achieves the lowest *SSE* and *AIC* values in most cases compared to the other models. We thus find that the RD-ARD model outperforms the other models, as is demonstrated by having a lower *AIC* score despite being penalized for having more parameters than the other models.

### Wave front speeds approximate population heterogeneity

A property of GBM spheroids is that the core and the invasive region do not expand at equal speeds. This has been observed in previous studies^26^, and in our dataset as well. This behavior is not described by the RD model, but is indeed captured by the RD-ARD model we propose. In order to study this behavior we propose a method (complete description in the “Traveling wave estimation” section) for calculating wave speed values at different cell densities, which in turn characterizes the spatial heterogeneity in the cell population. In short, we numerically calculate two different wave propagation speeds at two distinct cell densities. The corresponding wave speeds are denoted by ${c}_{\min }$ and ${c}_{\max }$. As we will show, these together with the difference ${c}_{{\rm{diff}}}={c}_{\max }-{c}_{\min }$ provide insights into the heterogeneity in cell population, or in other words, the deviation away from a homogeneous single population Fisher-KPP model. We also define the parameter *c*_shape_ to be the slope of the line passing through the densities corresponding to ${c}_{\max }$ and *c*_min_.

**Fig. 4:**
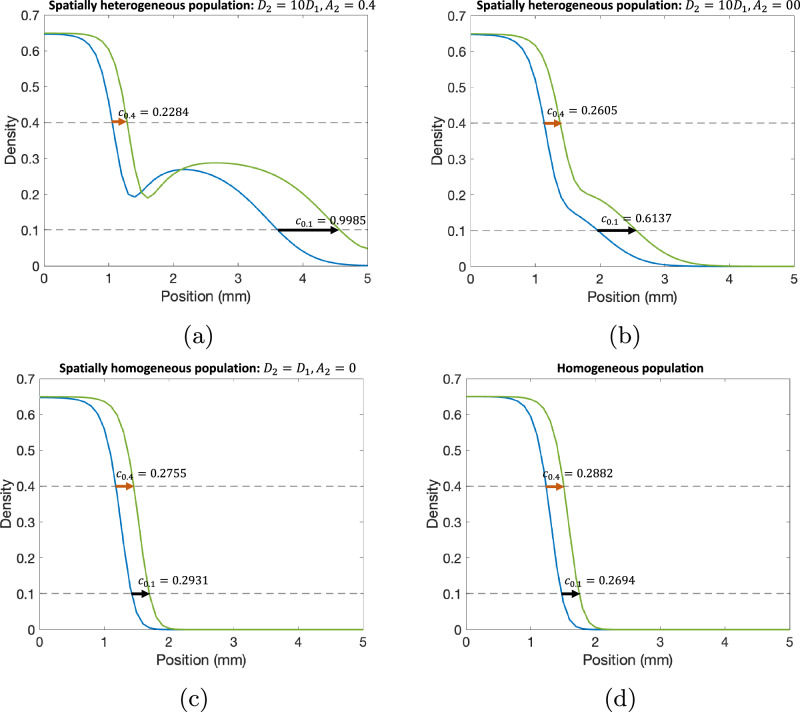
Visualization for wave front speeds at different densities for different scenarios. **a** A spatially heterogeneous population with *D*_2_ = 10*D*_1_ and *A*_2_ = 0.4, **b** a spatially heterogeneous population with *D*_2_ = 10*D*_1_ and *A*_2_ = 0, **c** a spatially homogeneous population with *D*_2_ = *D*_1_ and *A*_2_ = 0, and **d** a homogeneous population. In these figures, we compare the wave front speeds at two different time points: (blue) *t* = 4.03 wk and (green) *t* = 5.00 wk. All parameter values for each simulation are presented in Table 2.

**Fig. 5:**
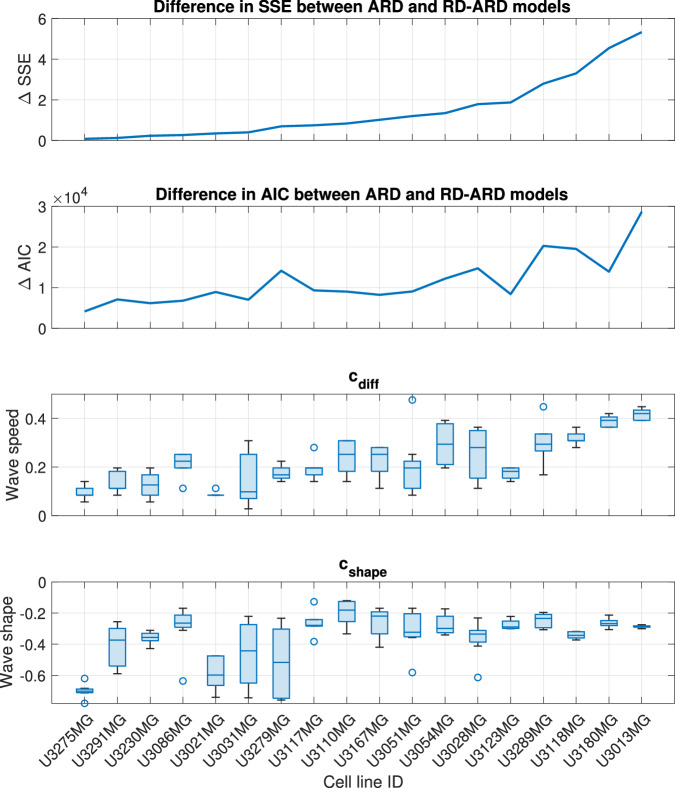
Visualization for the trends in Δ*SSE*, Δ*AIC*, *c*_diff_, and *c*_shape_. The cell lines are in an increasing order of Δ*SSE*.

We first sought to validate our proposed method for calculating wave speed values to characterize spatial heterogeneity using simulated data. We compared the approximated wave speeds at two different cell densities, as representations for densities at ${c}_{\max }$ and ${c}_{\min }$, for various cases from the RD-ARD model: (a) a spatially heterogeneous population with *D*_2_ = 10*D*_1_, *A*_2_ = 0.4; (b) a spatially heterogeneous population with *D*_2_ = 10*D*_1_, *A*_2_ = 0; (c) a spatially homogeneous population with *D*_2_ = *D*_1_, *A*_2_ = 0; and (d) a homogeneous population (Fig. 4). The full details for the parameter values for all cases are described in Table 2. For the spatially homogeneous population with *D*_2_ = *D*_1_, *A*_2_ = 0 in Fig. 5c, while both subpopulations have homogeneous migrating parameters (i.e., diffusion and advection coefficients are the same), their growth parameters are different. Thus, we refer to it as a spatially homogeneous population to distinguish it from the spatially heterogeneous populations in Fig. 4a, b and the homogeneous population in Fig. 4d, where there is only one population. The results demonstrate substantial differences in the computed wave speeds at high density compared to low density for spatially heterogeneous populations with distinct migration parameter values. For instance, in the case of *D*_2_ = 10*D*_1_ and *A*_2_ = 0.4, the approximated wave speed at density 0.4 is 0.2284, compared to 0.9985 at density 0.1. Similarly, for the spatially heterogeneous population with *D*_2_ = 10*D*_1_ and *A*_2_ = 0, the approximated wave speeds are 0.2605 and 0.6137 at the respective densities. In contrast, for a spatially homogeneous population with identical migration parameter values (*D*_2_ = *D*_1_ and *A*_2_ = 0), the approximated wave speeds are relatively close, as seen in Fig. 4c. The homogeneous population in Fig. 4d exhibits similar trends. The computed *c*_diff_ values are: (a) *c*_diff_ = 0.7701, (b) *c*_diff_ = 0.3532, (c) *c*_diff_ = 0.0176, and (d) *c*_diff_ = 0.0188. These findings demonstrate that our proposed method, utilizing wave speed, effectively characterizes population spatial heterogeneity, particularly when subpopulations have distinct migration parameter values, i.e., *D*_1_, *D*_2_, and *A*_2_.

**Table 2:** Parameter values used for simulation to validate wave speed as proxy for spatial heterogeneity

Parameter	Figure 4a	Figure 4b	Figure 4c	Figure 4d
*D*_1_	0.007	0.007	0.007	0.007
*D*_2_	0.07	0.07	0.007	0
*ρ*_1_	2.5	2.5	2.5	2.5
*ρ*_2_	1.5	1.5	1.5	0
*K*_1_	0.65	0.65	0.65	0.65
*K*_2_	0.4	0.4	0.4	0
*A*_2_	0.4	0	0	0
*α*	0.5	0.5	0.5	1

We applied our wave speed heterogeneity characterization approach to the best-fit simulations from each multicellular tumor spheroids experiment. Recall that we wish to determine whether *c*_diff_ is a good proxy for population spatial heterogeneity. To this end, we calculate the difference in *SSE* and *AIC* values between the ARD model and the RD-ARD model, which serve as representative models for spatially homogeneous and heterogeneous GBM populations, respectively. We use the difference in *SSE* and *AIC* values to represent spatial heterogeneity because these values quantify the added benefit of using a heterogeneous model to describe the data instead of a homogeneous model. We chose to use the ARD model, rather than the RD model, as the representative homogeneous model due to the lower *SSE* and *AIC* values of the ARD model compared to the RD model (refer to Fig. 3). In Fig. 5c, we present a boxplot illustrating *c*_diff_. The general trend observed in Fig. 5 across all three plots indicates an increase in spatial heterogeneity. This suggests that the difference in numerical wave speeds at different cell densities can effectively characterize spatial heterogeneity in GBM populations.

### A subset of cell lines exhibit Go-or-Grow behavior

**Fig. 6:**
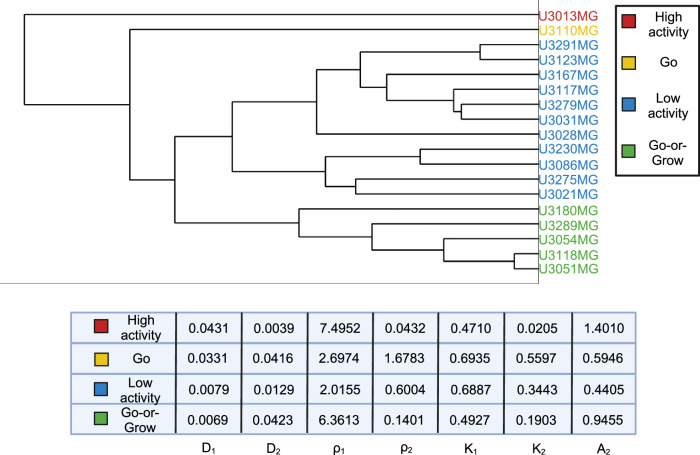
Dendogram visualizing the outcome of the hierarchical clustering. Coloring based on 4 clusters. The names are descriptive and chosen based on the properties of the cell lines within each cluster. The table shows the parameter averages for each cluster.

**Fig. 7:**
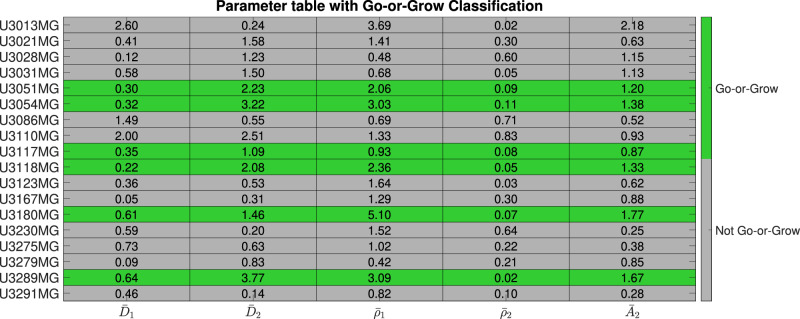
Parameter table with Go-or-Grow classification. Table of the fitted parameters after normalizing with respect to population averages, and the Go-or-Grow classification obtained from the criteria in (10) and (11).

In order to better understand the similarities and differences between the cell lines, we performed hierarchical clustering using MATLAB (MATLAB’s built-in function linkage with “average” method and “Standardized Euclidean distance” metric) on the learned model parameters for each cell line. The resulting dendogram with 4 clusters is shown in Fig. 6: high-activity, “Go”, low-activity, and “Go-or-Grow” (see Supplementary Figures 1–4 for examples). We can see that in the first split, U3013MG constitutes the high-activity cluster, while all other cell lines make up the second group. This distinction arises from the unique nature of U3013MG within our experimental dataset. Within this cell line, the first population exhibits high diffusion and growth rates, while the second population has both low diffusion and growth rates but a high advection rate leading to the “hump” characteristic in Fig. 2. This sets the U3013MG cell line apart from the rest of the cell lines in our dataset. In the second group, we observe the separation between U3110MG (the “Go” cluster) and the two larger clusters. The U3110MG cell line is another unique case, where both subpopulations mainly focus on migrating without proliferating. This is because the cluster is characterized by high and similar diffusion rates and low and similar growth rates. Additionally, the advection rate for the second subpopulation is also low compared to others. This explains why the *SSE* and *AIC* curves overlap in Fig. 3 at the U3110MG cell line, and the difference in *SSE* between the ARD and RD-ARD models is relatively low (see Fig. 5). When looking at the next split that occurs when four clusters are present, we can see that the the previous large cluster is split in such a way that U3180MG, U3289MG, U3054MG, U3118MG and U3051MG constitute the fourth cluster. By manually inspecting the learned model parameters (complete set of learner parameters are provided in Supplementary Table 2) we notice two things. The first is that most cell lines in cluster three exhibit low activities with low diffusion, proliferation and advection. The second thing we notice is that the cell lines of cluster four have the property that one subpopulation is dominated by migration (low growth rate and high diffusion and advection rates) and the other subpopulation by growth (high growth rate, low diffusion rate), resulting in the “hump” characteristic. We therefore form the hypothesis that this cluster can be characterized as a “Go-or-Grow” cluster. We test our hypothesis by applying our Go-or-Grow characterization method (see the section “Go-or-Grow classification”) to the best-fit parameters from each cell line (Fig. 7). We found that a total of 6 of the 18 cell lines are classified as Go-or-Grow (U3051MG, U3054MG, U3117MG, U3118MG, U3180MG, U3289MG). Five of these exactly match those making up cluster four obtained from the hierarchical clustering. The only cell line we classified as Go-or-Grow that is missing from cluster four is U3117MG. These results support the notion that a subset of cell lines can be described as exhibiting Go-or-Grow behavior. We can see that the cell lines which were not classified as Go-or-Grow have other characteristic behaviors. For example, some cell lines have a “weak” Go-or-Grow phenotype, but the relationship is not as strong (i.e., the Go-or-Grow criteria would be satisfied for smaller *k*). This is the case for U3021MG, U3031MG, U3123MG, and U3167MG. In one case we saw both migration and proliferation of comparable magnitude in both subpopulations (U3110MG). An outlier to these classes of behavior is the cell line U3013MG. The first subpopulation is both diffusing and proliferating, while the second subpopulation is dominated by the advective term. This behavior is not seen in any of the other cell lines. It is worth noticing that this cell line is one where we notice the largest improvement in model fit compared to a single population model, as can be seen in Fig. 3.

### Correlation between patient outcome and mathematical model parameters

We proceed to investigate if the best-fit RD-ARD model parameters correlate with patient age and survival. Of the 18 patient-derived cell lines we used in our work, two (U3123MG, U3291MG) were excluded on the basis that the patients were still alive at the time of creating the Human Glioma Cell Culture (HGCC) biobank, and hence have no associated actual survival time.

**Fig. 8:**
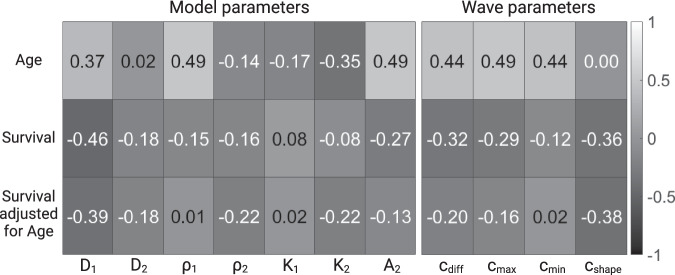
Correlation between model parameters and patient age and survival. Correlation between model parameters and age (row 1), between model parameters and survival (row 2) and between model parameters and age-adjusted survival (row 3).

We calculate the correlation between the parameters and patient age at diagnosis (row 1, Fig. 8), as well as between parameters and patient survival (row 2, Fig. 8). Since it has previously been established that survival is influenced by age at diagnosis^43–45^, we also calculated the partial correlation between model parameters and survival, adjusting for age (row 3, Fig. 8). For three variables, *A*, *B*, and *C*, the correlation between A and B, controlling for C is calculated via:

$${\rho }_{A,B\cdot C}=\frac{{\rho }_{A,B}-{\rho }_{A,C}{\rho }_{B,C}}{\sqrt{1-{\rho }_{A,C}^{2}}\sqrt{1-{\rho }_{B,C}^{2}}}.$$

where *ρ*_*A*,*B*_ is the correlation between *A* and *B*.

Since the number of patients is relatively small, it is difficult to interpret the results in a definite manner. However, since the correlation between age at diagnosis and survival is well established for GBM patients, we calculated it in our dataset, and found it to be −0.46. This can be used as a reference to compare the other correlation coefficients against.

Our analysis reveals that the model parameters are generally most strongly correlated with patient age (*D*_1_, *ρ*_1_, *A*_2_, *c*_diff_, ${c}_{\max }$, ${c}_{\min }$). When looking at how the parameters are correlated with survival, both with and without adjusting for age, we see that *D*_1_ is the most strongly correlated with survival, followed by *c*_shape_.

## Discussion

This study combined tumor spheroid experiments, microscopy imaging, mathematical modeling, and data analysis methods to develop and analyze a novel data-driven PDE model for in vitro tumor spheroid growth and invasion. We performed 136 total tumor spheroid experiments from 18 separate cell lines and quantified the radial spheroid density of each experiment over time using signal processing methods. To better interpret this data, we compared four mathematical models of tumor spheroid growth: the RD, ARD, RD-RD (or 2-population RD), and RD-ARD models. In the majority of cases the RD-ARD model outperformed the three other PDE models (quantified with the Akaike Information Criterion under the framework of least square) in describing data from the 18 cell lines. A traveling wave analysis characterized the spatial heterogeneity of the RD-ARD model and can be used to determine parameter values where this model behaves differently from the homogeneous models. A clustering analysis on the estimated parameters from the RD-ARD model allowed us to group together cell lines with similar parameter estimate values.

Population heterogeneity and advection are assumed in the RD-ARD model, allowing it to capture the “hump” characteristic observed in some of the cell lines, as shown in Fig. 2d. Figures 2 and 3 have demonstrated the importance of including not only the population heterogeneity but also the advection term when modeling multicellular tumor spheroids. A key feature of the RD-ARD model is that a portion of the spheroid population migrates by advection while the entire population also migrates by diffusion. Advection refers to the directed movement of cells; in our model, cells are moving away from the spheroid center. Many previous studies have proposed that spheroid cells perform chemotaxis, or migration towards increasing chemoattractant chemical gradient levels. Possible chemoattractants for GBM cells include oxygen, glucose, CXCL8, CXCL12, transforming growth factor, epidermal growth factor, etc. A chemoattractant gradient away from the spheroid center forms in response to cell consumption. The authors of ref. ^46^ used a one-compartment PDE with density-dependent diffusion and chemoattractant-dependent chemotaxis to model glioma growth and invasion. This model agreed with previous experiments (and those in our study) that the outer invasive zone grows faster than the inner proliferative zone of the spheroid. In future work, it would be interesting to compare the performances of the RD-ARD model and the model from ref. ^46^ in fitting the spheroid data from our study.

Previous studies on the traveling wave speeds of tumor growth and invasion relied on the PDE modeling framework with a single population, using the RD model, i.e., the Fisher-KPP equation. This assumption of a single population allows for the analytical computation of the minimum traveling wave speed constant and the wave shape-related ratio. While these analytically computed wave parameters have been found to be clinically relevant, computing them for models with heterogeneous populations poses a challenge. Instead, we numerically computed the wave parameters and proposed that they can be used to characterize spatial heterogeneity in the population. We observed a general trend where the approximated wave parameters increase as the *SSE* and *AIC* values increase, indicating that these approximated wave parameters can potentially be used to characterize spatial heterogeneity.

The RD-ARD model assumes that one population migrates and proliferates, whereas the second population, in addition also exhibits motion due to advection. An advantage of this model is that it is very flexible and, as such, can be used to describe many different population behaviors. For example, it is possible that both subpopulations act similarly (e.g., both invade and grow fast or slowly) or distinct from each other (e.g., one primarily invades while the other primarily grows). Having defined a criteria for Go-or-Grow, we proposed that 6 of the 18 cell lines appear to exhibit the Grow-or-Go behavior, in which one subpopulation primarily grows while the other predominantly invades. Other cell lines exhibited a weaker Grow-or-Go phenotype or similar behavior between the two subpopulations. These vast differences in parameter estimates between cell lines highlights the wide variation in MCTS dynamics from our data. This indicates the need for precision medicine tools to determine the most effective treatment strategies for each type of population behavior found with our modeling framework. The parameter *c*_shape_ quantifies the shape of the invasive front. A smaller number indicates a more diffuse spheroid, and a larger value a spheroid with a sharp boundary. This parameter is obtained numerically and plays a role similar to that of the ratio *D*/*ρ* from the Fisher-KPP equation.

Our correlation analysis showed that a number of model parameters are correlated with patient age at diagnosis, and *D*_1_ and to a lesser extent *c*_shape_, with patient survival. Although one always wishes for stronger correlations, we find the results encouraging. The fact that we found a correlation between *D*_1_ and survival, similar in magnitude to that of age and survival, is encouraging and warrants further research with a larger number of patient-derived cell lines. The reason for why *D*_1_ shows the strongest association with survival is a matter we can only speculate around. One possible hypothesis is that since it is the second subpopulation that is most often driven by diffusion, having a large *D*_1_ would correspond to a strongly invasive tumor spheroid, invading since both subpopulations (the entire tumor) have high motility.

In future work, we will investigate whether combining the modeling framework for MCTSs described here can provide an additional informative dimension to predicting the outcome of drug screens or interpreting gene expression data. For example, the estimated parameters from the RD-ARD model, simple metrics such as spheroid diameter as a proxy for growth^14,47^, and information from in vitro assays probing cytotoxicity^48^ or apoptosis^49^, can be concatenated into a feature vector for each patient-derived tumor cell line in order to predict drug efficacy. Alternatively, the estimated parameters from the RD-ARD model for a large set of patient-derived cell lines could be correlated with gene expression differences to provide hypotheses about which intracellular signaling pathways regulate phenotypes that affect spheroid dynamics, e.g., growth, diffusion, and advection. Other possible directions for future work include utilizing deep learning for data de-noising or automated model selection. In this study, we chose to use simulations generated by the optimized model parameters to compute the wave parameters due to the noise in the experimental data. For the future work, we propose employing neural networks for the data de-noising task. Neural networks have demonstrated superior performance in the presence of biologically realistic observation noise for the Fisher-KPP model^50^. This could potentially enable the computation of wave parameters without the need for parameter estimation and model simulation. Moreover, equation learning methods relying on neural networks have been implemented for automated model discovery in spatial-temporal data of reaction-diffusion equations^51,52^ and mean-field approximations of agent-based models for cell populations incorporating birth, death, and migration processes^22^. Despite the RD-ARD model exhibiting the best performance among the models discussed in this study, we observed that there still exists some model discrepancy for some cell lines. Equation learning could potentially be used to correct model discrepancy in these instances, enabling the ability to discover novel biophysical mechanisms governing the underlying dynamics of longitudinal MCTSs data.

## Methods

### Spheroid invasion assay

Patient-derived glioma cell cultures (GCCs) were chosen from the Human Glioma Cell Culture (HGCC) collection^53^. Each cell line was seeded into eight wells each of 96-well PrimeSurface S-BIO round bottom ultra-low attachment plates (MoBiTec), in defined serum-free neural stem cell (NSC) medium, supplemented with B-27, N2, EGF, and FGF as previously described^54^. A 50% matrigel (Corning, 356232, ≥8 mg/ml) overlay in defined NSC medium was added on day three and the spheres were allowed to invade into the extracellular matrix for six additional days. Starting from day three, images were acquired in the phase contrast and brightfield channels with the 4x objective every six hours, on an IncuCyte®S3 live-cell imaging system (Sartorius) using the spheroid software module version 2019B. The subtype classification and genomic profiles of all cell cultures used in this study have been previously described^55^. A total of 25 cell lines were originally included in the experiment, however, six were excluded because of lack of growth, and an additional cell line was excluded due to large amounts of noise when calculating the density profiles. The 18 cell lines, and the corresponding number of replicates for each can be found in Supplementary Table 1.

### Data processing

**Fig. 9:**
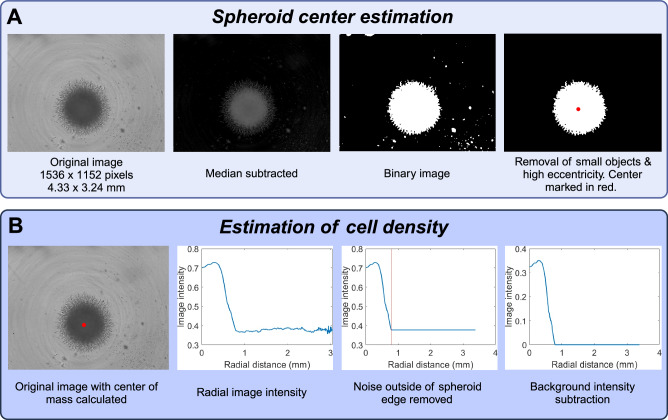
Data processing pipeline. The first step (**A**) is to estimate the location of the spheroid center. The second step (**B**) is to estimate cell density as a function of radial distance from the spheroid center. This figure was created with BioRender.com.

In order to estimate cell density profiles from the brightfield images (of size 1536 × 1152 pixels, and 4.33 × 3.24 mm), we developed custom software in MATLAB. The goal is to convert 2D images of spheroids to radial density profiles. The data processing therefore consists of two distinct problems: (i) finding the center of mass of the spheroid, (ii) converting image intensity to cell density. The major steps are shown in Fig. 9.

The process of estimating the center of mass is illustrated in Fig. 9 and consists of the following steps:

1. Background subtraction,
2. Binarization, and
3. Removal of small objects.

We then proceed to estimate cell density as a function of radial distance to the spheroid center. This process consists of the following steps.

1. Calculation of image intensity as a function of radial distance to the spheroid core,
2. Removing noise outside the spheroid boundary, and
3. Background subtraction.

Note that the intensity profiles obtained after processing are referred to as cell densities. These are not normalized to have values in any particular range. We found that they typically range between 0 and 0.5. The best case would be to be able to obtain the actual cell number, but this is a challenging problem, and to the best of our knowledge, is not possible to do from 2D projected images of 3D cultures.

**Algorithm 1.** Pseudocode for estimation of spheroid center of mass

 Input image *im*_0_;

* m* = **median**(*im*_0_);

* im*_subtracted_ = *im*_0_ − *m*;

* im*_smoothed_ = **imgaussfilt**(*im*_subtracted_, 4);

* im*_binary_ = **im2bw**(*im*_smoothed_, 0.08);

* ConnComp* = **bwconncomp**(*im*_binary_);

** for** *object in ConnComp* **do**

 | If *object* has area < 4000 pixels^2^, remove it from *im*_binary_;

**end**

 Delete all objects in *im*_binary_ except the one with the smallest eccentricity (most round shape) ;

 Find all rows **r** and columns **c** where *im*_binary_ = 1;

 Center of mass = (**mean**(**r**), **mean**(**c**));

The function names written in bold are built-in MATLAB functions. We first subtract the median intensity from the original image, as a form of background subtraction. The function **imgaussfilt**(*im*_intensity_, 4) applies a Gaussian filter with standard deviation 4. The function **im2bw**(*im*_smoothed_, 0.08) binarizes the image based on the threshold 0.08. The function **bwconncomp**(*im*_binary_) finds the connected components (i.e., distinct cohesive objects) in the binary image. We then remove objects smaller than 4000 pixels^2^. This corresponds to an area of around 32,000 μm^2^. For reference, this is half the size of a typical spheroid before it starts growing. Having deleted small objects, we continue to delete all remaining objects except for the one with lowest eccentricity (most rounded shape). This step is performed due to certain images having large objects from the background noise remaining after the previous steps. However, they are typically not rounded, and can hence be removed by this step. Having a single rounded object remaining in the image (the spheroid) we compute the center of mass by taking the mean of the non-zero row indices and the non-zero column indices.

**Algorithm 2.** Pseudocode for estimation of radial cell density

 Input image *im*_0_ and center of mass of the spheroid;

* im*_intensity_ = 1 − *im*_0_;

** for** each pixel in *im*_intensity_**do**

 | Calculate distance between *pixel* and spheroid center of mass;

**end**

 Discretize the space into 600 bins of size 2 pixels. Put all pixels into their respective bins. Calculate average intensity for each bin. We obtain an array **y**_*i*_, *i* = 1, …, 600, of length 600, with values between 0 and 1;

 Calculate simple moving average **y**_*avg*_ of the density **y**;

 Estimate the boundary of the spheroid by finding the first point i* where the derivative of the moving average changes sign;

 Set the intensity to be constant after *i*. ${y}_{j}={y}_{{i}^{* }}$, for *j* ≥ i*;

 Remove background intensity by subtracting $y=y-{y}_{{i}^{* }}$

The discretization into bins of size 2 pixels helps to reduce noise. The maximum distance of 1200 pixels (600 bins) was chosen to guarantee that the entire spheroid was captured. In order to detect the spheroid boundary we found the first inflection point where the derivative changed from negative to positive. This can be challenging when the data is noisy, and for that reason we took two additional measures to ensure its accuracy. First we used the simple moving average (100 bins on either side of the given point), which handles noise much better. Second, we look for points to the right of bin 100, which ensures that we ignore points inside the tumor core. The final steps of the processing is to set the intensity to be constant from the spheroid boundary and outward, and then to subtract it from the density, so that the surrounding region has density 0. The resulting image intensities are the ones we use as a proxy for cell density throughout our paper. They typically attain values between 0 and 0.5, but are not normalized to lie in any particular range.

### Parameter estimation method

In order to perform parameter estimation, we first need to numerically solve a PDE model to approximate the true model solution. In this work, we implement central differencing to approximate the first-order and second-order derivatives in space. We then use the MATLAB built-in function ode15s to numerically integrate the model. During the parameter estimation step, the algorithm minimizes the sum of squares error (*SSE*) to find the most optimal set of parameters, $\hat{{\boldsymbol{q}}}$. The optimized parameters are determined from

5 $$\hat{{\boldsymbol{q}}}=\mathop{{\mathrm{arg}}\,{\mathrm{min}}}\limits_{{\boldsymbol{q}}\in {\boldsymbol{Q}}}\mathop{\sum }\limits_{i,j}^{{N}_{t},{N}_{r}}{\left[{u}_{i,j}^{o}-\hat{u}({t}_{i},{r}_{j};{\boldsymbol{q}})\right]}^{2},$$

where ${u}_{i,j}^{o}$ and $\hat{u}({t}_{i},{r}_{j};{\boldsymbol{q}})$ are the observed and simulated total cell density at the *i*th temporal point and *j*th spatial radial location. In addition, *N*_*t*_ and *N*_*r*_ are the total number of temporal points and spatial locations in the data. ***Q*** denotes the set of admissible values for the model parameters with the parameter bounds being shown in Table 3 along with model parameters information. We select the parameter ranges for the diffusion coefficient and growth rate based on the patient-reported values in the previous study^16^. We converted Wang et al. estimated values^16^ from mm^2^/year and year^−1^ to mm^2^/week and week^−1^. We then choose the appropriate upper bounds to be within an order of magnitude of their estimated values. Similar values of *D* and *ρ* in mm^2^/year and year^−1^ also have been used to learn equations of simulated data generated using the Fisher-KPP equation with one dimensional spatial data^52^.

**Table 3:** Parameter descriptions and ranges

Parameter	Description	Range	Unit
*D*_1_	Population 1 diffusion coefficient	0–0.2	mm^2^/week
*D*_2_	Population 2 diffusion coefficient	0–0.2	mm^2^/week
*ρ*_1_	Population 1 growth rate	0–15	week^−1^
*ρ*_2_	Population 2 growth rate	0–15	week^−1^
*K*_1_	Population 1 carrying capacity	0–1	cells/mm^3^
*K*_2_	Population 2 carrying capacity	0–1	cells/mm^3^
*A*_2_	Population 2 advection rate	0–3	mm/week
*α*	Proportional of initial condition belonging to population 1	0–1	Unitless

As a result of the non-linearity in the proposed model, using a local optimization algorithm might not guarantee the most optimal results. To perform parameter estimation, we first implement a global optimization algorithm called the DIRECT (DIviding RECTangles) algorithm^56^. DIRECT is a derivative-free global optimization algorithm that works by dividing all hyper-rectangles of parameter space into smaller hyper-rectangles to find the optimal hyper-rectangle, that is, the hyper-rectangle with the smallest error at the center. While DIRECT is fast to approach the area surrounding the global minimum, it can be slow to converge to the global minimum with high accuracy^57^. In this work, we use the DIRECT algorithm as the initial step for parameter estimation. In the second step, we use the optimized parameters estimated by the DIRECT algorithm as the initial starting values for a local optimization algorithm. The DIRECT algorithm is implemented using a MATLAB package provided in ref. ^58^. For the local algorithm, we use fmincon, a MATLAB built-in function, with interior-point as the algorithm.

### Model comparison

To appropriately assess model performance, the use of the *SSE* for comparison might not be sufficient, as it fails to consider the inherent complexity of the models. Since the RD-ARD model is the most complex model, i.e., having more parameters than the other models, it is expected to outperform the other models with the *SSE* metric. To account for the complexity of the model, we rely on the Akaike Information Criteria (*AIC*)^59,60^, which penalizes complex models with more parameters. The *AIC* under the framework of least squares problem was developed by Banks and Joyner^60^ and is described as follows:

6 $$AIC={N}_{t}{N}_{r}\left[\ln (2\pi )+1\right]+{N}_{t}{N}_{r}\ln \left(\frac{SSE}{{N}_{t}{N}_{r}}\right)+2({\kappa }_{{\boldsymbol{q}}}+1),$$

where *N*_*t*_ and *N*_*r*_ are the total number of temporal and spatial points, *SSE* is the sum of squares error described as a sum in Eq. (5), and *κ*_***q***_ is the total number of parameters being estimated.

### Traveling wave estimation

Many previous PDE frameworks utilized single compartment PDE models, such as the Fisher-KPP equation, to describe tumor spheroid growth and invasion. A common pattern that arises in these models is a traveling wave, or a stationary spatial profile that moves at a fixed speed (referred to as the wave speed). For example, the diffusion coefficient *D* and the growth rate *ρ* can be approximated by identifying the minimum traveling wave speed constant, $2\sqrt{D\rho }$, and the wave shape related ratio (or relative invasiveness ratio), *D*/*ρ*^16–18,21^. These parameters are then used to classify patient tumors into different categories: slow (low *D* and low *ρ*), diffuse (high *D* and low *ρ*), nodular (low *D* and high *ρ*), and fast (high *D* and high *ρ*)^16–18,21,52^. While this approach has been found to be clinically relevant^16–18^, it is based on the assumption of intratumoral homogeneity. In addition, MCTSs comprised of GBM cells often have a core region and an invasive region where cells appear to exhibit different behavior. In particular, the core region expands at a slower speed compared to the invasive region^26^. Single compartment models which admits a traveling wave solution fail to capture this behavior. On the other hand, two-compartment models lacking traveling wave solutions can be analyzed by tracking the propagation speed at different cell densities, which is what we do in this study.

We choose two total cell density values *u*^(1)^ and *u*^(2)^, and numerically (following refs. ^23,24^) calculate the speed with which both values propagate. Throughout the paper we will refer to these as wave speeds, even though they are not technically traveling waves. In models admitting traveling waves, the two wave speeds are equal, whereas in spatially heterogeneous two-compartment models they generally are not. We propose that the difference in propagation speeds at different cell densities is a good proxy for intra-tumor spatial phenotypical heterogeneity.

#### Algorithm 3

**Numerically approximated wave speed computation algorithm** 
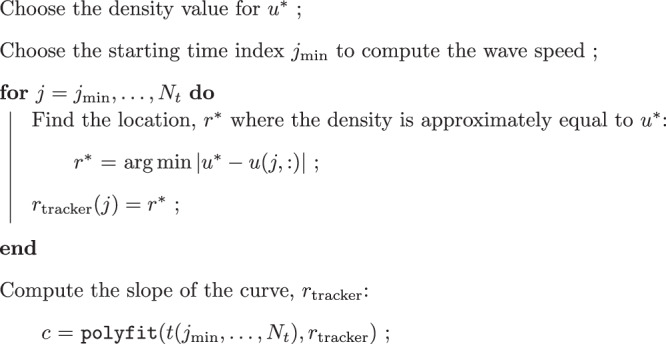


**Fig. 10:**
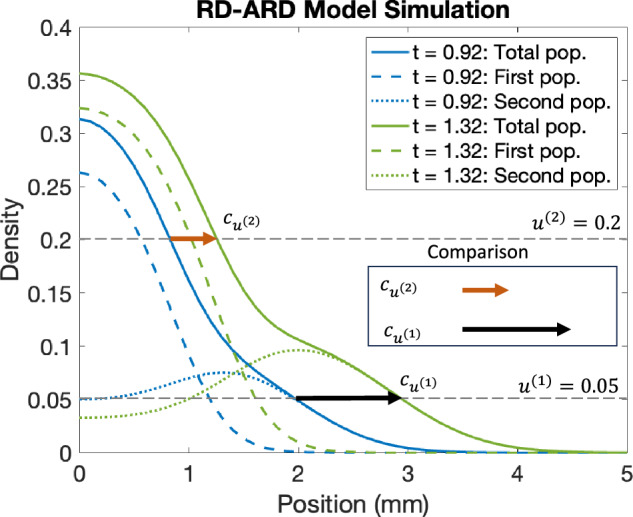
A representative figure for comparing wave speeds at different densities. The dashed blue and green curves are the cell density for the first population at two different time points, *t*_*i*_ (blue) and *t*_*f*_ (green) with *t*_*i*_ < *t*_*f*_. Similarly, the dotted blue and green curves are the cell density for the second population. The solid blue and green curves are the total cell density. The orange and black arrows represent the wave speed vectors at *u*^(1)^ = 0.2 and *u*^(2)^ = 0.05. As being shown, the front density at 0.2 travels at slower speed compared the the front density 0.05. This shows that the speed of the density profile depends on the chosen density threshold, *u*^(*i*)^, utilized in calculating the wave speed.

An example simulation of a spatially heterogeneous population is shown in Fig. 10, and illustrates the idea behind our proposed approach, for *u*^(1)^ = 0.05 and *u*^(2)^ = 0.2. In Fig. 10, we observe that the wave speed for *u*^(1)^ is greater than the wave speed for *u*^(2)^. The difference in wave speed is due to how the higher density profile is primarily affected by the first population, whereas the lower density profile is influenced by both the first and second populations. This indicates that, the front of density moves faster where the density is low compared to where it is high. In other words, in a spatially heterogeneous population, the speed of the wavefront changes depending on the specific density threshold, *u*^(*i*)^, being used to compute the wave speed.

While in Fig. 10, we choose to visualize the difference in wave speeds at *u*^(1)^ = 0.05 and *u*^(2)^ = 0.2, in practice, it is challenging to predetermine the exact density values for comparison, because cell lines exhibit different density profiles. This means a cell lines might have different maximum density values and densities value at the “hump” characteristic. Therefore, we compute the wave speed at different cell densities and compare the difference between the maximum and minimum computed wave speeds. Let {*u*^(1)^, *u*^(2)^, …, *u*^(*M*−1)^, *u*^(*M*)^} be the set of predetermined cell densities where we compute the wave speeds. We then define

$$\begin{array}{ll}{c}_{\max }\,=\,\max \{{c}_{1},\ldots ,{c}_{M}\},\\ {c}_{\min }\,=\,\min \{{c}_{1},\ldots ,{c}_{M}\},\\ {c}_{{\rm{diff}}}\,=\,{c}_{\max }-{c}_{\min },\end{array}$$

where *c*_1_, …, *c*_*M*_ are the wave speeds at cell densities *u*^(1)^, …, *u*^(*M*)^, respectively. In addition, another challenge that could arise is to decide which the time points for which we compute the wave speeds. We choose to compute the wave speed using a linear regression-based method over all time points, as suggested in previous studies^23,24^. For each density value *u*^(*i*)^, we track the location *x* where the density is approximately equal to *u*^(*i*)^ over time. The approximated wave speed at total density *u*^(*i*)^ is the slope of the curve formed by the tracked *r* values over time. We use the MATLAB function polyfit to compute the slope of the best fit line between time and the location of *u*^(*i*)^ over time. In other words, for each total density threshold *u*^(*i*)^, we track the list of *t*^(*i*)^ and *r*^(*i*)^ such that $u\left({t}^{(i)},{r}^{(i)}\right)={u}^{(i)}$. The curve formed by the tracked points $\left({t}^{(i)},{r}^{(i)}\right)$ is then fitted using a linear line with the form *r* = *c*_*i*_*t* + *b*, where *c*_*i*_ is the approximated wave speed at *u*^(*i*)^. The pseudocode for the numerically approximated wave speed computation is described in Algorithm 3. We also define the parameter *c*_shape_ to be the slope of the line passing through the densities corresponding to ${c}_{\max }$ and ${c}_{\min }$ at the final time point. We will refer to these four parameters as wave parameters throughout this paper.

In a study conducted by Stepien et al., a numerical method was utilized to calculate the wave speed for a model that admits a stable traveling wave solution^23^. The stability of the traveling wave solution is reflected by a wave profile that maintains a constant speed once the tumor core has stabilized at its maximum cell density. Consequently, after reaching this maximum density, the wave speed can be determined numerically from the solution. Because the collected data did not reach the maximum cell density during the data collection process, we simulated the RD-ARD model with the estimated parameters from each dataset and utilized the cell density data from *t* = 0.75*t*_*f*_ to *t* = *t*_*f*_, where *t*_*f*_ is the final time value to calculate the wave speed for each cell line. We computed the wave speeds at 100 cell density values ranging from 0.02 to 0.8${u}_{\max }$ for each data set, with ${u}_{\max }$ being the maximum density value in the data set. The maximum and minimum of the approximated wave speeds were then used for comparison. The reason to compute the wave parameters simulations was driven by the experimental data’s inherent noise, which could significantly influence computed results. Simulations enable us to denoise the data before proceeding with the computation of wave parameters.

### Go-or-Grow classification

Our RD-ARD model can be regarded as a Go-or-Grow type model, whenever one subpopulation is predominantly migrating and the other proliferating. In a model of RD-RD (or 2-population RD) type where no advection is present, one can check if the diffusion coefficient of one subpopulation is significantly larger than that of the other subpopulation, while the roles of the proliferation terms are reversed. For our model we need to take the advection term into account, since it can contribute to the motility of the second subpopulation. There is no straightforward way to compare the motility resulting from advection to that resulting from diffusion. We chose to do it by normalizing each coefficient with the population-average, rendering each coefficient dimensionless. We first calculate the population averaged coefficients:

$$\begin{array}{rcl}\bar{D}&=&\mathop{\sum }\limits_{i=1}^{18}\frac{{D}_{1}^{i}+{D}_{2}^{i}}{2},\\ \bar{\rho }&=&\mathop{\sum }\limits_{i=1}^{18}\frac{{\rho }_{1}^{i}+{\rho }_{2}^{i}}{2},\\ \bar{A}&=&\mathop{\sum }\limits_{i=1}^{18}{A}_{2}^{i}.\end{array}$$

For cell line *i*, with fitted model parameters $\{{D}_{1}^{i},{D}_{2}^{i},{\rho }_{1}^{i},{\rho }_{2}^{i},{K}_{1}^{i},{K}_{2}^{i},{A}_{2}^{i}\}$ we next normalize the fitted coefficients with the population averages:

7 $${\bar{D}}_{1}^{i}=\frac{{D}_{1}^{i}}{\bar{D}},\qquad {\bar{D}}_{2}^{i}=\frac{{D}_{2}^{i}}{\bar{D}},$$

8 $${\bar{\rho }}_{1}^{i}=\frac{{\rho }_{1}^{i}}{\bar{\rho }},\qquad {\bar{\rho }}_{2}^{i}=\frac{{\rho }_{1}^{2}}{\bar{\rho }},$$

9 $${\bar{A}}_{2}^{i}=\frac{{A}_{2}^{i}}{\bar{A}}.$$

A model is classified as being of Go-or-Grow type if it satisfies one of the conditions:

10 $${\bar{D}}_{1}\, >\, k\cdot ({\bar{D}}_{2}+{\bar{A}}_{2})\,{\text{and}}\,{\bar{\rho}}_{2}\, >\, k\cdot {\bar{\rho }}_{1},$$

11 $$({\bar{D}}_{2}+{\bar{A}}_{2})\, >\, k\cdot {\bar{D}}_{1}\,{\text{and}}\,{\bar{\rho }}_{1}\, >\, k\cdot {\bar{\rho }}_{2},$$

where *k* is a multiplicative factor that governs how much larger the migration and proliferation of one population must be, relative to the other population. The first condition ensures that the first subpopulation is mainly migrating and that the second subpopulation is mainly proliferating, and the second condition reverses the roles for the two subpopulations. We use *k* = 5 as a criteria, meaning that the rescaled migration and proliferation is at least five times larger in one subpopulation compared to the other.

## Supplementary information

Supplementary Information

## Data Availability

The raw data (cell density profiles) can be made available upon request.

## Code availability

The code is publicly available at: https://github.com/kcnguyen3191/Modeling-Tumor-Spheroids.
