# Supplementary material for: Mathematical modeling of multicellular tumor spheroids quantifies inter-patient and intra-tumor heterogeneity
Source: NPJ Syst Biol Appl. 2025 Feb 15;11:20. doi: 10.1038/s41540-025-00492-3 (PMC11830081; doi:10.1038/s41540-025-00492-3)
Supplement: Supplementary file 1 — Supplementary Information [file 41540_2025_492_MOESM1_ESM.pdf]

Supplementary Information: Mathematical Modeling of Multicellular Tumor Spheroids Quantifies Inter-Patient and Intra-Tumor Heterogeneity

Adam A. Malik*[1], Kyle C. Nguyen*[2,3], John T. Nardini[4], Cecilia C. Krona[6], Kevin B. Flores[3,5], and Sven Nelander[6]

[1]Mathematical Sciences, Chalmers University of Technology, Gothenburg, Sweden.
[2]Biomathematics Graduate Program, North Carolina State University, Raleigh, NC, USA
[3]Center for Research in Scientific Computation, North Carolina State University, Raleigh, NC, USA
[4]Department of Mathematics and Statistics, The College of New Jersey, Ewing, New Jersey, USA
[5]Department of Mathematics, North Carolina State University, Raleigh, NC, USA
[6]Department of Immunology, Genetics and Pathology, Uppsala University, Uppsala, Sweden.

* Equal contribution
Corresponding author: maadam@chalmers.se

Supplementary Information

Cell-line	Replicates	Patient age at diagnosis	Patient Survival (days)
U3013MG	8	78	122
U3123MG	4	64	1961
U3118MG	7	57	793
U3180MG	8	80	267
U3289MG	8	75	486
U3051MG	8	72	493
U3054MG	8	60	611
U3279MG	8	56	333
U3031MG	8	65	468
U3167MG	8	58	1234
U3291MG	8	60	1527
U3117MG	6	57	793
U3110MG	7	58	243
U3021MG	6	50	387
U3028MG	8	72	496
U3230MG	8	61	712
U3275MG	8	74	206
U3086MG	8	72	444
**Total**	**136**		

Supplementary Table 1: Cell line information and replicate count

Cell-line	D_1	D_2	ρ_1	ρ_2	K_1	K_2	A_2
U3013MG	0.0431	0.0039	7.4952	0.0432	0.4710	0.0205	1.4010
U3110MG	0.0331	0.0416	2.6974	1.6783	0.6935	0.5597	0.5946
U3291MG	0.0076	0.0023	1.6756	0.2003	0.6250	0.2047	0.1788
U3123MG	0.0059	0.0088	3.3321	0.0690	0.5893	0.1157	0.3982
U3167MG	0.0008	0.0051	2.6258	0.6197	0.7784	0.4760	0.5628
U3117MG	0.0058	0.0181	1.8872	0.1682	0.8121	0.3450	0.5562
U3279MG	0.0014	0.0137	0.8627	0.4356	0.9270	0.1697	0.5472
U3031MG	0.0096	0.0248	1.3823	0.0937	0.9260	0.1245	0.7241
U3028MG	0.0020	0.0203	0.9769	1.2226	0.9734	0.1339	0.7363
U3230MG	0.0097	0.0033	3.0817	1.2962	0.4292	0.4815	0.1588
U3086MG	0.0247	0.0091	1.4038	1.4414	0.4881	0.4289	0.3325
U3275MG	0.0121	0.0104	2.0786	0.4551	0.6669	0.7612	0.2443
U3021MG	0.0068	0.0262	2.8638	0.6024	0.3604	0.5460	0.4065
U3180MG	0.0101	0.0241	10.3756	0.1474	0.4931	0.0935	1.1372
U3289MG	0.0107	0.0625	6.2843	0.0433	0.4291	0.0350	1.0732
U3054MG	0.0053	0.0533	6.1552	0.2319	0.4315	0.3039	0.8888
U3118MG	0.0036	0.0344	4.8046	0.1033	0.6156	0.2269	0.8564
U3051MG	0.0050	0.0370	4.1869	0.1745	0.4942	0.2921	0.7721

Supplementary Table 2: Fitted model (RD-ARD) parameters. The units of the diffusion coefficients are mm^2/week, the growth rates $week^{-1}$, and the advection mm/week.

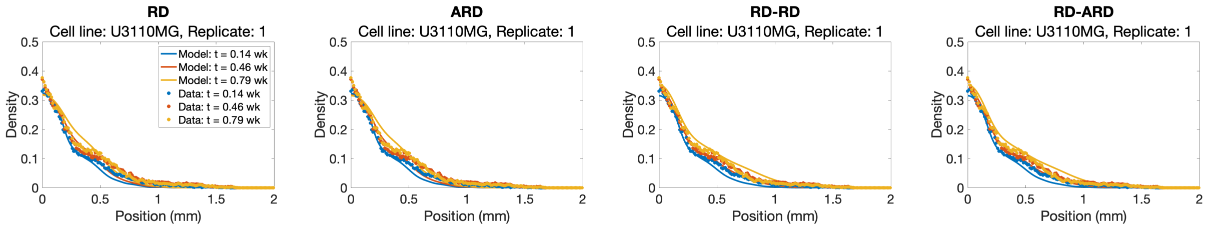

Supplementary Figure 1: A visualization of fitting results for the **"Go"** cluster using 4 models (from the left-most column to the right-most column): (a) RD, (b) ARD, (c) RD-RD (or 2-population RD), and (d) RD-ARD models for a representative replicate of cell lines **U3110MG**. We plot the total cell density data against the location for different time point as dotted color curves. The solid color curves represents the model fittings. Time is measured in weeks.

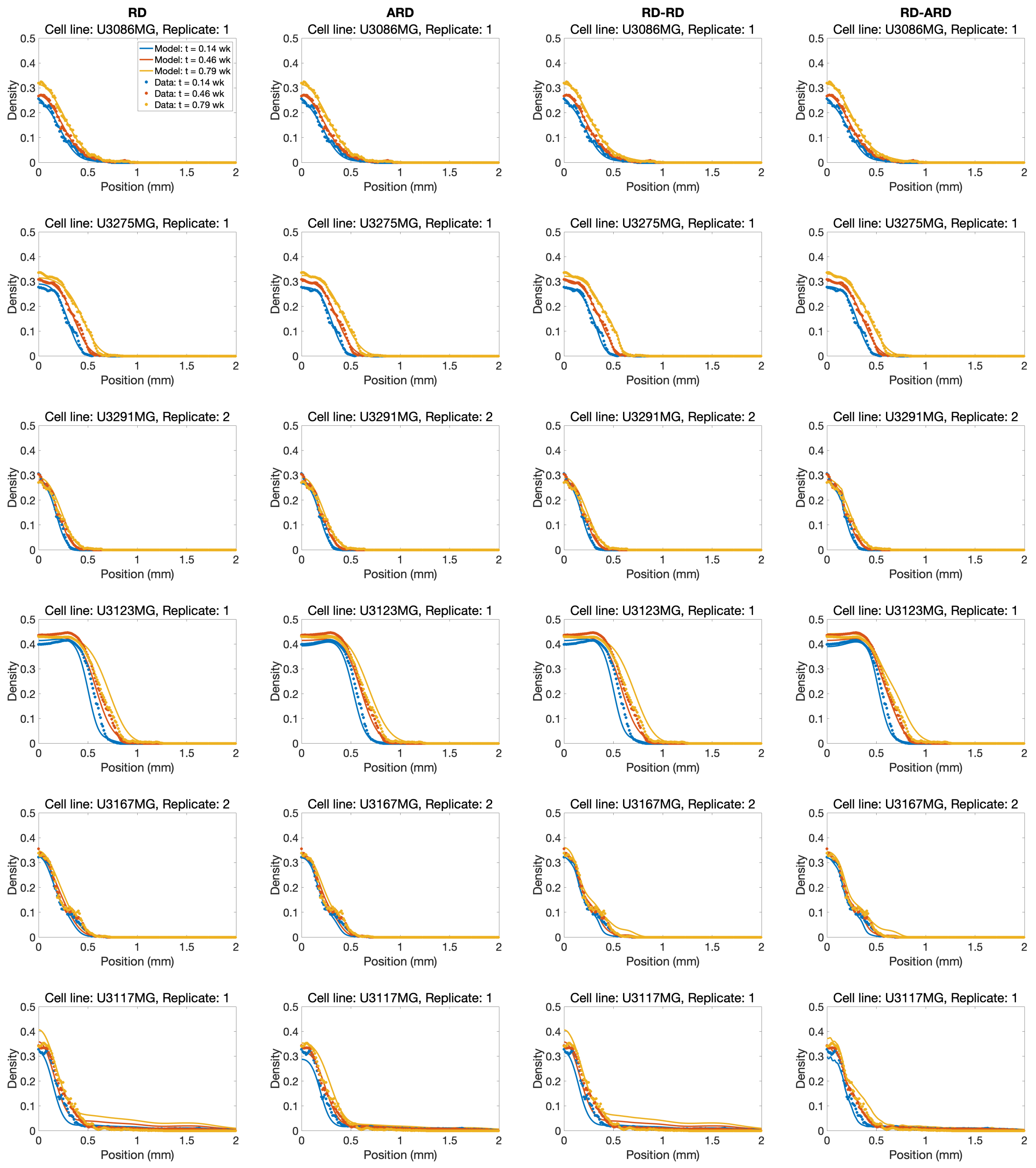

Supplementary Figure 2: A visualization of fitting results for the **"Low-activity"** cluster using 4 models (from the left-most column to the right-most column): (a) RD, (b) ARD, (c) RD-RD (or 2-population RD), and (d) RD-ARD models for representative replicates of cell lines **U3086MG, U3275MG, U3291MG, U3123MG, U3167MG,** and **U3117MG**. We plot the total cell density data against the location for different time point as dotted color curves. The solid color curves represents the model fittings. Time is measured in weeks.

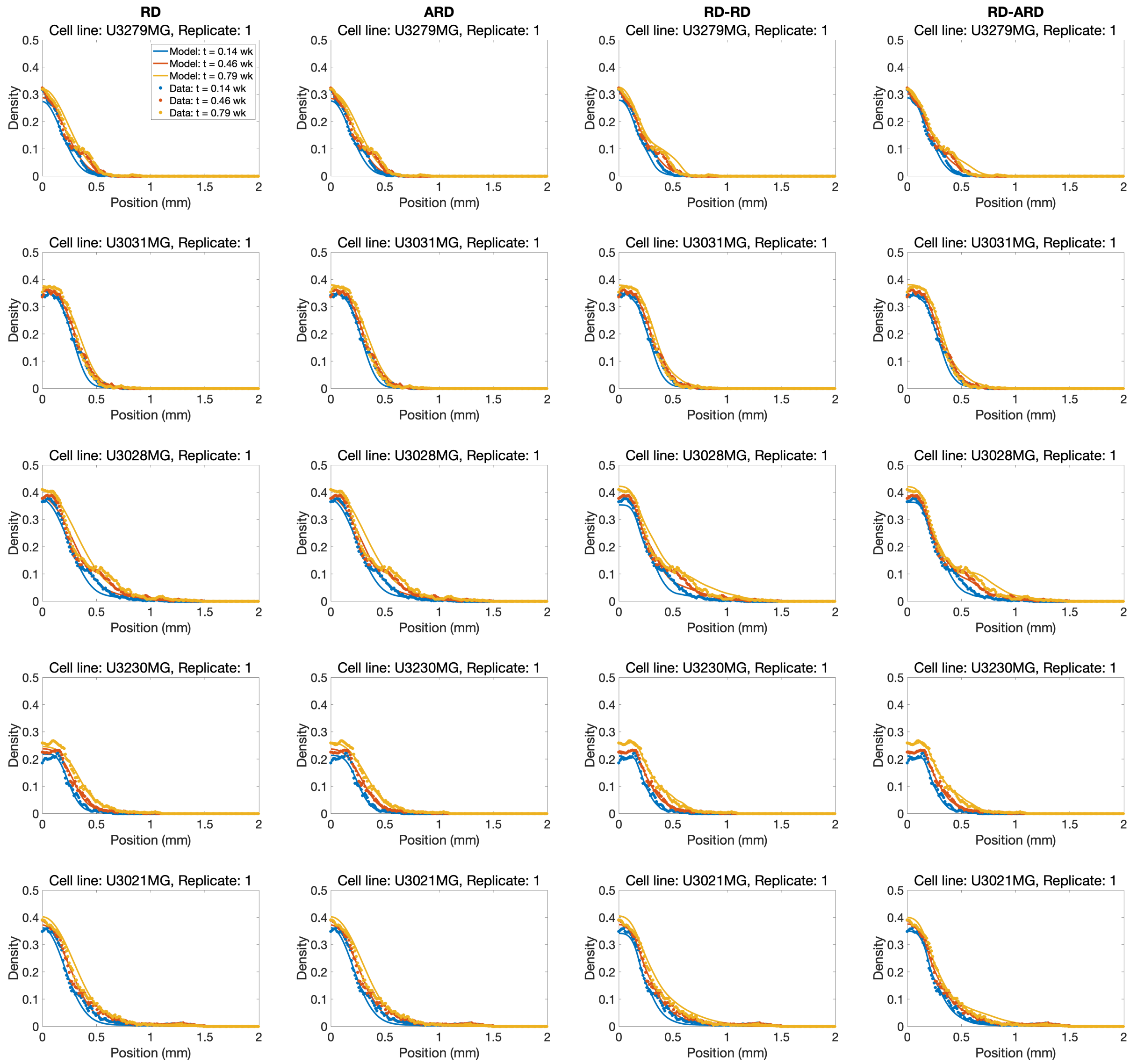

Supplementary Figure 3: A visualization of fitting results for the **“Low-activity”** cluster using 4 models (from the left-most column to the right-most column): (a) RD, (b) ARD, (c) RD-RD (or 2-population RD), and (d) RD-ARD models for representative replicates of cell lines **U3279MG, U3031G, U3028MG, U3230MG,** and **U3021MG**. We plot the total cell density data against the location for different time point as dotted color curves. The solid color curves represents the model fittings. Time is measured in weeks.

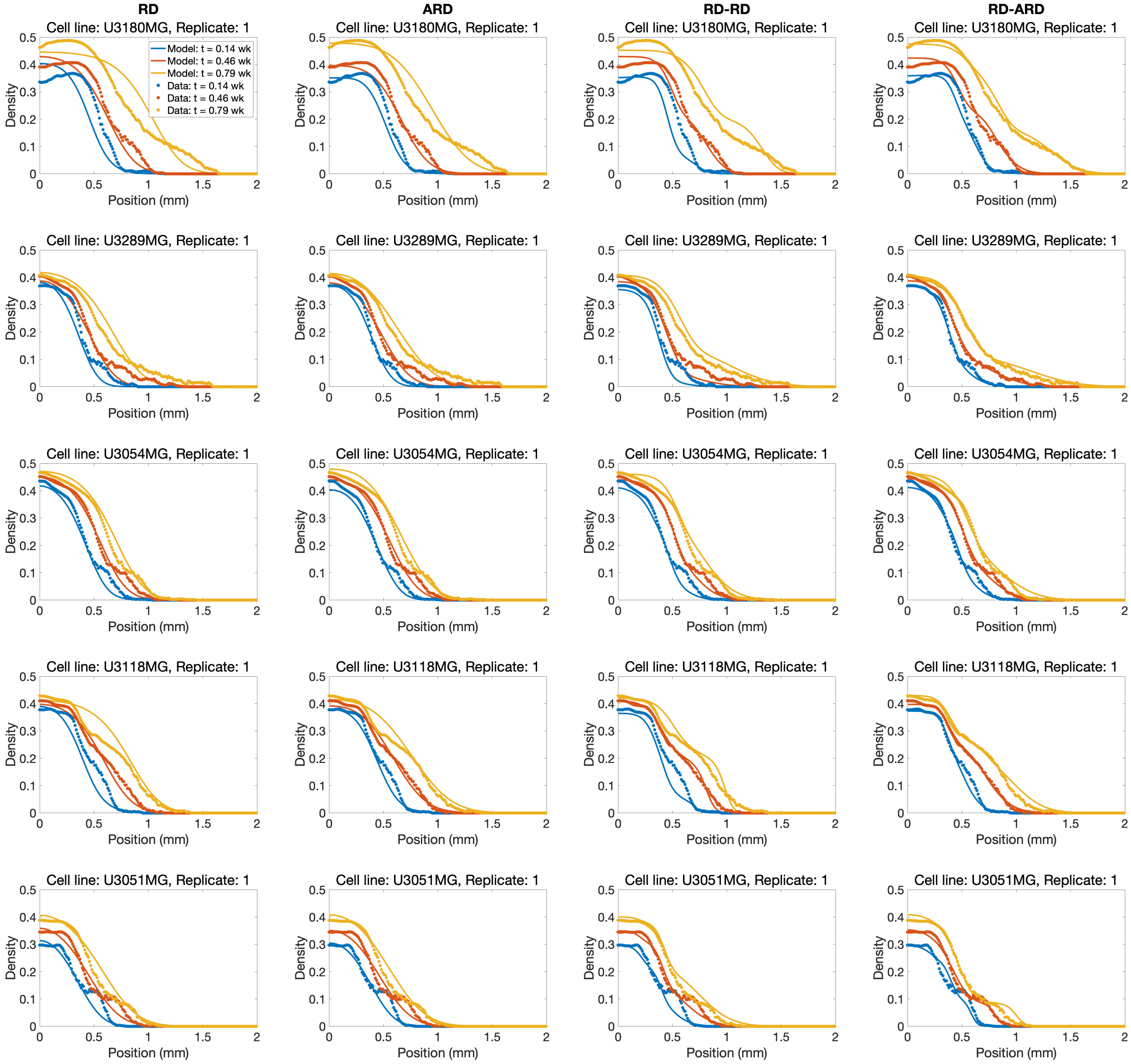

Supplementary Figure 4: A visualization of fitting results for the **"Go-or-Grow"** cluster using 4 models (from the left-most column to the right-most column): (a) RD, (b) ARD, (c) RD-RD (or 2-population RD), and (d) RD-ARD models for representative replicates of cell lines **U3180MG, U3289MG, U3054MG, U3118MG**, and **U3051MG**. We plot the total cell density data against the location for different time point as dotted color curves. The solid color curves represents the model fittings. Time is measured in weeks.
